# History in your hand

**DOI:** 10.5195/jmla.2021.1316

**Published:** 2021-10-01

**Authors:** Stephen J. Greenberg

**Affiliations:** 1 patzere4@gmail.com, Retired

**Keywords:** history, archival management, COVID-19, race and gender studies

## Abstract

In the swirl of current events including a pandemic and new chapters in the awareness of race and gender, it is the professional responsibility of librarians and archivists to create durable records for future scholars, so they can understand our present.

We've learned a lot of new words and lessons in the last year or so (remember when the word “zoom” was pretty much limited to graphic novels, Marvel movies, and kids' games?). We learned about the possibilities (and limitations!) of a socially distanced, digitally empowered, professional world. Now, as a new, postpandemic world appears to be an actual possibility, we almost have time to catch our collective breath and look back. It's not the end, but then again, history doesn't stop; it's one damned thing after another, as Arnold Toynbee said (maybe). And that's exactly the point. There's never a good time to stop and reflect. You need to look back as you move forward.

So much of this was new, unexpected, and far beyond “strange” to so many of us. To others, it was a series of new variations on abiding themes. The answers weren't actually different, but they fit new questions with remarkable ease. It was the questions that needed refining.

Take, for example, the use of surrogates to access texts from shuttered library collections. Suddenly, digital copies were all we had. This was a shock to some parts of the information universe, but it should not have been. Born-digital and solely digital content are still the minority of what's out there. For the last decade or so, we had come to see the paper version as the backup to the digital. But that's not what happened, when the paper was beyond reach. And that should not have been a surprise. The first commercially viable surrogate was microfilm, which was initially marketed as a way to back up a vulnerable paper collection. The Army Medical Library (AML), known these days as the National Library of Medicine (NLM), began microfilming its journal collection in the 1930s as a hedge against destruction during wartime. I looked but failed to find evidence that copyright was considered to be a barrier to filming. To use modern terminology, the AML was creating a “dark archive,” to be accessed only in the case of the destruction of the originals by enemy action. This thinking (which also led to the evacuation of the AML rare book collection to Cleveland in 1942–1943) was remarkably prescient. The catalog of the British Library still notes items destroyed by Nazi air raids. It was only after the war that microfilm (and its even nastier cousins microfiche and microcard) were seen as commodities to be sold in grand sets around the world. Companies such as University Microfilm International (once a division of Bell & Howell, which made microfilm hardware) found their market share and grew up to be ProQuest. Nobody ever liked using microfilm; in an interview in the 1980s, when microfilm was seen as the best hedge against brittle, acidic paper, historian Barbara Tuchman said she absolutely hated it, except that sometimes, it was indispensable [1]. We all know a lot of things like that, I expect. But something much greater is at play here.

I can write all this easily enough because I have more or less permanent records to consult: a printed history of NLM by Wyndham Miles [2], the archival records of the AML held by NLM and the National Archives [3, 4], documents from UMI in the 1980s stating their corporate connections [5], and a DVD of Tuchman's interview as part of the classic film documentary *Slow Fires: On the Preservation of the Human Record* [1]. Added to this is a layer of internalized oral history (also known as “memory”) of wrestling with scratched service copy film on dirty microfilm readers in cold library basements in the Bronx or Lower Manhattan on wet November nights. “Old men forget,” but the records will remain if only we keep them.

And that is the point here. It is never too soon to remember. The time to create records is now; it is always so. Archiving the evidence of what has happened over the last year and a half cannot wait. Our local institutions need not be concerned about the grand events; the National Archives and the *New York Times* (and Fox News) will handle those. But preserving the records of what happened in our own backyards—our reading rooms and hospital wards and campuses—that is our responsibility.

One day (although it seems surreal to think about it), there will be a COVID-19 Centennial. What will those future historians have from us? Tony Fauci, for sure, but elbow bumps and the shortage of toilet paper? We can do more than that. We can give them more than that.

It is also important, in fact, vitally so, to realize how lucky we are to be able to create this record for the future. The past months have been about more issues than just the pandemic (although it does seem strange to write that). We have all been reminded how dreadfully easy it has been to deny or ignore the history of others based on their color, gender, ethnic background, or sexual identification. In the United States, generations of human beings were forbidden their history, their languages, their cultures, their identities. A certain level of awareness cannot be eradicated, but there are limits to memory, especially when you are denied your own language, and equally cut off from the ability to read and write in another. Because these tools were banned, we can never know the dimensions of all that was lost.

The denial of a people's right to exist, and to own their history, cannot be allowed to stand, much less go forward. Historians who know the power of their tools are already at work, using every technique at their disposal to recover whatever they can. The anniversaries of Stonewall and the Tulsa Race Massacre have given new impetus to reexamining what evidence can be recovered. The task grows harder the further back in time one looks, but the task itself is not impossible. And it is a task that can no longer be delayed.

For this denial to be avoided in a shared future, there is another task that cannot be delayed, and it is the task alluded to earlier. Now is the time to remember, when you hold your history in the palm of your hand. Memory is fresh; write it down, record it, digitize it, keep it safe. Have your institution do the same. Good records-management procedures are nice to follow, and there is plenty of free guidance available on the web from the Society of American Archivists and other groups, but you don't need a professional just to remember. Get the raw data down and keep it safe; the finding aids can wait.

In the pages of this journal, in a somewhat more jocular context, I once quoted the so-called “Compulsory Preface” of *1066 and All That*, by W.C. Sellar and R.J. Yeatman from 1931, but eerily relevant for where we stand right now: “History is not what you thought. *It is what you can remember*. All other history defeats itself.” The emphasis in the original [6].

Now is the time to remember, when your history is in your hand. You owe it to your ancestors, to your descendants, and, most especially, to yourself.
